# Attitude and preventive practices towards COVID-19 disease and the impact of awareness training on knowledge of the disease among correctional officers

**DOI:** 10.35241/emeraldopenres.13839.2

**Published:** 2022-01-21

**Authors:** Johnson Okoro, Ambrose Ekeroku, Benedicta Nweze, Tobechukwu Odionye, Joel Nkire, Martins Onuoha, Chinenye Ezeonwuka, Jude Owoh

**Affiliations:** 1 Nigerian Correctional Service, Enugu, Enugu State, Nigeria; 2 Carmelite Prisoners Interest Organization, Enugu, Enugu State, Nigeria; 3 Federal Neuropsychiatric Hospital, Enugu, Enugu State, Nigeria; 4 Nigerian Correctional Service, Yola, Adamawa, Nigeria; 5 Project Development Institute, Enugu, Enugu State, Nigeria; 6 Biological Sciences, Quinnipiac University, Connecticut, CT, United States

**Keywords:** COVID-19, Knowledge, Attitude, Practice, Correctional officers

## Abstract

COVID-19 remains a public health emergency of international concern. Efforts at the global and national levels are being made to control its spread. The Nigerian Correctional Service is also proactive in the fight against the disease by organizing COVID-19 awareness training for correctional officers. We conducted a pre- and post-test assessment of COVID-19 knowledge among correctional officers in Enugu State Command to determine the impact of awareness training on their knowledge level. The study also assessed correctional officers’ attitude and preventive practices towards the disease.

The mean knowledge score was 19.34 out of 25, and the awareness training significantly improved the participants’ COVID-19 knowledge. We found a significant moderate, positive correlation between knowledge and attitude/practice, and a significantly higher knowledge level among those with higher educational qualifications.

Regular hand washing with soap and water (87.9%), wearing face masks (84.4%), and social distancing (83%) were practiced by the majority of the participants. . The majority of the participants (53.2%) received COVID-19 information from multiple sources including the Nigeria Centre for Disease Control and the World Health Organization

## Introduction

COVID-19 is a novel viral disease discovered in Wuhan, Hubei Province, China in 2019 and is caused by severe acute respiratory syndrome coronavirus 2 (SARS-CoV-2) ( Chen *et al.*, 2020). The index cases of the disease were as a result of animal to human transmission. However, human-to-human transmission from respiratory droplets and contact with contaminated surfaces has been implicated in subsequent cases ( Adhikari *et al.*, 2020; Hassan *et al.*, 2020; Li *et al.*, 2020).

The symptoms of this highly contagious disease include dry cough, fever, anosmia (loss of smell), weakness, headache, body pains, vomiting, sore throat, and respiratory difficulty, and the onset of symptoms ranges from 1–14 days ( Ahmed, 2020; Wang *et al.*, 2020). Some infected individuals may remain asymptomatic (without symptoms) after contracting the virus ( Lee *et al.*, 2020; Oran & Topol, 2020), whereas among the symptomatic cases, the majority of them are mild or moderate ( Bi *et al.*, 2020; Chang *et al.*, 2020), with about 10% being severe ( Bi *et al.*, 2020; Chang *et al.*, 2020).

While all age groups can equally contract the virus, the elderly are more vulnerable. Other vulnerability factors are obesity, underlying medical conditions such as diabetes mellitus, systemic hypertension and other cardiac pathologies, and immune-compromising diseases such as HIV infection ( Ahmed, 2020; Chen *et al.*, 2020; Guan *et al.*, 2020; Huang *et al.*, 2020; Zhang *et al.*, 2020; Zhou *et al.*, 2020). Similarly, mortality rate has been reported to be higher among these categories of people ( Guan *et al.*, 2020; Zhang *et al.*, 2020).

Considering that there was no treatment or vaccine available against the disease during the early period of its outbreak ( Ahmed, 2020), preventive measures appeared to be the only scientific evidence available to curtail the high spread and mortality associated with it. These measures included practices such as regular hand washing with soap and water, the use of alcohol-base hand sanitizer, wearing face masks in public places, avoiding crowded places, and maintaining social distancing ( Chu *et al.*, 2020; WHO advice for the public).

The level of knowledge of a disease condition is associated with attitude towards the disease, and these interact to substantially affect the practices and measures aimed at controlling it ( Ayinde *et al.*, 2020; Choi & Yang, 2010; Hung, 2003; Yap *et al.*, 2010; Zhong *et al.*, 2020). One study in Pakistan that examined medical students’ knowledge, perceptions, and behavioral intentions towards the H1N1 influenza observed that inadequate knowledge and a negative attitude are associated with poor compliance with practices designed to prevent the spread of the disease ( Hussain *et al.*, 2012). In other studies, it was also found that adequate knowledge propels individuals to comply with practices and measures that promote good health ( Aziz *et al.*, 2018; Rahman & Sathi, 2020).

More so, availability of information, the source of information and demographic variables such as gender and educational level have a great effect on people’s knowledge, behavioral response and compliance towards the necessary preventive measures against a disease outbreak ( Ahmed *et al.*, 2020; Al-Hazmi *et al.*, 2018; Ayinde *et al.*, 2020; Erfani *et al.*, 2020; Olum *et al.*, 2020; Tandi *et al.*, 2018; Zhong *et al.*, 2020).

Knowledge of the COVID-19 disease has been acquired through several channels, with different social media platforms and the internet dominating as the major sources of information of this novel disease ( Abdelhafiz *et al.*, 2020; Ahmed *et al.*, 2020; Alzoubi *et al.*, 2020; Huynh *et al.*, 2020). Studies have documented robust evidence that people who obtained their information through professional and scientific institutions or personnel have a positive attitude and higher confidence about the disease condition than those who obtained information from informal sources such as friends and relatives ( Tandi *et al.*, 2018).

Given that adequate knowledge and a positive attitude towards COVID-19 among correctional officers is essential in effective control and prevention of disease outbreak in the prison population, the appropriate steps in this regard should entail an assessment of their knowledge, attitude and practice towards the COVID-19 pandemic. To this end, the Nigerian Correctional Service and the Carmelite Prisoners’ Interest Organization (CAPIO) organized a three-day awareness training to educate all correctional officers in Enugu State Command. The awareness education was delivered by a team led by a medical doctor and covered several areas including case identification, and infection prevention and control. While correctional officers’ knowledge, attitude and preventive practices towards COVID-19 were assessed before the commencement of the training, knowledge level was also evaluated after the training to determine the impact of the training on the participants.

Therefore, our study was guided by the following objectives:

1. To determine the baseline knowledge level, attitude and preventive practices of correctional officers towards COVID-19
2. To evaluate the impact of COVID-19 awareness training on their knowledge level
3. To assess the association between socio-demographic characteristics, and attitude and knowledge at baseline
4. To assess the correlation between knowledge, attitude and practice

## Methods

### Ethical considerations

Permission to carry out this study among correctional officers was obtained from the Nigerian Correctional Service with reference number ES/EP.124/Vol.11/30, which was approved on 4 ^th^ June, 2020. Formal ethical approval was not obtained because the study design was of low risk nature, in which the only foreseeable risk on the participants was the time they spent filling the forms, and this is in line with the Nigerian Code of Health Research Ethics.

The objectives of the study were explained to the participants before the commencement of the awareness training. They were made to understand that participation in the study was voluntary. It was further explained to them that they could withdraw from the study at any stage even after giving consent, and that such withdrawal or not giving consent would not in any way stop them from attending the training. Thereafter, verbal and written consent were taken.

### Study design and setting

This was an interventional study with a pretest and post-test assessment to evaluate the impact of COVID-19 awareness training among all correctional officers of Enugu State Command. Enugu State Command of the Nigerian Correctional Service has three lock-up custodial centers situated in Enugu metropolis, Oji and Nsukka.

This was a three-day training that was held on 8 ^th^, 11 ^th^ and 12 ^th^ of June, 2020; with each day dedicated to one lock-up center such that officers of Nsukka, Enugu, and Oji custodial centers were trained on 8 ^th^, 11 ^th^, and 12 ^th^ of June 2020, respectively. The awareness program was organized by the Nigerian Correctional Service and the Carmelite Prisoners’ Interest Organization (CAPIO). This program was facilitated by the consultant psychiatrist and head of the medical department of the Nigerian Correctional Service, Enugu State Command, other mental health professionals (psychiatrists and psychologists) and research experts from the CAPIO. The topics addressed during the training comprised: : symptoms of COVID-19, epidemiology and risk factors of COVID-19, disease transmission, and guidelines and preventive measures against it. Correctional officers of the three correctional facilities in Nsukka, Oji, and Enugu metropolis were involved. Each of these custodial centers has a lecture hall where the program was conducted.

### Participants

Of the 156 correctional officers from the three lock-up custodial centers that were trained, 141 of them completed the pretest assessment while 134 completed the post-test assessment. The post-test participation was reduced by 15 as some of the participants were recalled by the prison authority to their sensitive security duty post; hence, they were not present at the end of the training during which the post-test questionnaire was delivered.

### Inclusion criteria

Those working in the three lock-up custodial centers that received the awareness training and gave consent to participate.

### Exclusion criteria

Staff who came late for the training missed the lecture; hence, they were excluded from the study.

### Variables

The independent variables were the socio-demographic variables while the dependent variables were COVID-19 knowledge and attitude.

### Measurement

Our study used a self-reported questionnaire ( Okoro *et al.*, 2020d) which was divided into two sections and was administered before and after the awareness training. The first section covered the participants’ socio-demographic characteristics, while the second section was about knowledge, attitude, and practices towards COVID-19 disease. The socio-demographic section contained questions about age, educational level, gender and sources of COVID-19 information. Knowledge related questions were guided by the surveys of previous studies ( Abdelhafiz *et al.*, 2020; Olum *et al.*, 2020), as well as information from the World Health Organization health topics on coronavirus. A total of 25 questions covering four domains of symptoms, prevention, epidemiology and transmission were used to assess the participants’ knowledge of COVID-19. The options were “yes,” “no,” or “I don’t know.” For every correct answer, one point was assigned; while a wrong or I don’t know response attracted zero points. Therefore, the total knowledge score ranged from 0–25, where a higher score was indicative of a greater knowledge of the disease.

The four attitude and the four practice related questions were adaptations of previous studies ( Olum *et al.*, 2020; Rahman & Sathi, 2020; Zhong *et al.*, 2020). Participants were asked to choose a “no,” or “yes” response to the practice questions. Zero points were assigned to a non-practice and one point to each preventive practice. Hence, the total practice score ranged from zero to four, with a higher score indicating greater compliance with preventive practices. Participants were asked to choose “yes,” “no” or “not sure” to the attitude questions. Zero points were assigned to a “no” or “not sure” response, while one point was assigned to a “yes” response. A higher score indicates a positive attitude while a lower score indicates a negative attitude.

All the questionnaires used in this study were in English language.

### Data processing

A frequency check was run on the obtained data to check for any missing data. The distribution of the continuous data was checked using the Kolmogorov-Smirnov test. Age, pre-test knowledge, and post-test knowledge data were normally distributed (P>0.05). Therefore, parametric statistical tools were used for the analyses.

### Statistical analyses

The IBM Statistical Package for Social Sciences (IBM SPSS) statistical software, version 20 was used for analyses. A paired t-test was used to summarize the pretest and post-test knowledge level of the participants. Partial correlation statistics was employed to test the correlation between knowledge level and practice, while controlling for attitude. Test of association was further done using an independent t-test, chi-squared test, and ANOVA where appropriate. Statistical significance was set at P < 0.05.

## Results

Table 1 shows the socio-demographic characteristics of the participants and their associations with attitude.

**Table 1.** Socio-demographic characteristics of the participants (N=141).

Variables	Those that believe there are cases in Nigeria			Those that believe there are cases in the world			Those that believe there will be successful control			Those that believe there will be victory		
	n(%)	*x ^2^*	p	n(%)	*x ^2^*	p	n(%)	*x ^2^*	p	n(%)	*x ^2^*	p
**Gender** F(n=30) M(n=111)	27(19.9) 109(80.1)	4.64	0.07	28(20.7) 107(79.3)	0.54	0.61	29(22) 102(78)	0.82	0.69	26(21) 98(79)	0.06	0.76
**Source** W/N(n=44) Sf(n=22) xple(n=75)	43(31.6) 18(13.2) 75(55.2)	16.74	<0.01	41(30.4) 20(14.8) 74(54.8)	3.54	0.17	42(32.1) 20(15.3) 69(52.7)	0.66	0.72	40(32.3) 18(14.5) 66(53.2)	1.14	0.56
**Education** pry(n=9) sec(n=31) 3rd(n=101)	7(5.1) 30(22.1) 99(72.8)	9.91	0.01	7(5.2) 30(22.1) 98(72.6)	0.62	0.02	7(5.3) 29(22.1) 95(72.5)	3.35	0.19		12.96	0.01
		t-test			t-test			t-test			t-test	
**Age (yrs)** 39.28±9.18		0.23	0.82		0.61	0.55		-0.06	0.95		-0.55	0.58

n=number, F=female, M=male, W/N=WHO/NCDC, sf=social media/friends, xple=multiple, pry=primary, sec=secondary, 3 ^rd^=tertiary, yrs=years, *x ^2^* =chi-squared test, p=p-value.

A total of 141 participants completed the pretest assessment. The majority of them were males (111, 78.7%) and had tertiary education (101, 71.6%) with a mean age of 39.28±9.18 ( Okoro *et al.*, 2020b). More than half of the participants (75, 55.2%) reported that their major source of information was through multiple means, while for 43 (31.6%), their major source of information was from the World Health Organization or the Nigeria Center for Disease Control (NCDC). Among those who believed that there are confirmed cases of COVID-19 in Nigeria, 43 (31.6%), 18 (13.2%) and 75 (55.2%) received their information from the WHO website or NCDC website/text messages; social media/friends; and multiple sources, respectively. The association between information source and belief that there are confirmed cases of COVID-19 in Nigeria is statistically significant (p˂0.01). A statistically significant association was also found between educational qualification and those that believed there are cases in Nigeria, those who believed that there are cases in other parts of the world, and those who believed that the world will win the fight against the virus. 

Table 2 shows the association between knowledge and socio-demographic characteristics. Higher educational qualification was significantly associated with a higher knowledge of the disease. Other demographic characteristics showed no significant association with knowledge.

**Table 2.** Association between knowledge and demographic characteristics.

		Frequency	Knowledge score	t/F	p-value
**Gender**	Female	30	19.50±3.57	0.264	0.792
	Male	111	19.30±3.78		
**Education**	Primary	9	14.22±3.90	15.891	˂0.001
	Secondary	31	18.03±82.0		
	Tertiary	101	20.20±3.19		
**Source of information**	WHO	7	18.86±4.14		
	NCDC	37	19.62±2.86	1.580	0.197
	Sf	22	17.82±4.69		
	Multiple	75	19.69±3.71		
**Age (years)**	30 and below	18	18.56±4.00	0.910	0.405
	31–40	65	19.15±3.68		
	above 40	58	19.79±3.69		

Sf=social media/friend, NCDC=Nigeria Center for Disease Control, WHO=World Health Organization.

Table 3 shows the baseline knowledge level of the participants in four domains, namely symptoms, preventive measures, means of spread, and epidemiology. The total knowledge score ranged from 9 to 25, with a mean of 19.34±3.72. Knowledge about the preventive practices were very high, such that almost all the participants 140 (99.3%) correctly answered that regular hand washing with soap and water is a way of preventing the disease. Similarly, 136 (96.5%), 136 (96.5%), and 134 (95.0%) agreed that avoiding crowded places, wearing face masks when leaving home, and the use of alcohol base hand sanitizers, respectively, are ways of preventing the disease. The lowest level of knowledge was for questions on the presence of a vaccine/drug, eating of wild animals as a possible source of the disease, and loss of smell as a symptom, in which the respective figures were 66 (46.8%), 78 (55.3%), and 56 (39.7%).

**Table 3.** Baseline knowledge of COVD-19 among participants (N=141).

COVID-19 knowledge items	No/I don’t know	Yes
	n (%)	n (%)
**Symptoms include**		
Fever	11(7.8)	130(92.2)
Cough	17(12.1)	124(87.9)
Weakness	54(38.3)	87(61.7)
Body pain and headache	42(29.8)	99(70.2)
Breathing difficulty	51(36.2)	90(63.8)
Sore throat	52(36.9)	89(63.1)
Vomiting	29(20.6)	112(79.4)
Loss of smell (anosmia)	85(60.3)	56(39.7)
**Preventive measures include**		
Regular hand washing with soap and water	1 (0.7)	140(99.3)
Use of alcohol-based hand sanitizer	7 (5.0)	134(95.0)
Avoiding going to crowded places	5(3.5)	136(96.5)
Wearing a face mask in public places	5(3.5)	136(96.5)
Coughing into bent elbow or tissue and immediately discarding it	15(10.6)	126(89.4)
Keeping distance of at least 1 meter from people	10(7.1)	131(92.9)
Quarantining new inmates for 14 days	23(16.3)	118(83.7)
Quarantining close contacts of a confirmed case	10(7.1)	131(92.9)
Isolating and treating confirmed cases	21(14.9)	120(85.1)
**It can be spread by**		
Eating wild animals	63(44.7)	78(55.3)
Respiratory droplets	39(27.7)	102(72.3)
Touching contaminated surfaces and touching the mouth/eyes/nose	27(19.1)	114(80.9)
**Epidemiology includes**		
Most cases are not severe	70(49.6)	71(50.4)
Old age and underlying medical conditions like Diabetes and HIV are risk factors	30(21.3)	111(78.7)
Children and adults can equally be infected	42(29.8)	99(70.2)
Symptom onset is from 1–14 days	28(19.9)	113(80.1)
There is a known vaccine or drug for treating it	75(53.2)	66(46.8)
**Total score** Min-Max Mean±S.D	9–25 19.34±3.72	

Table 4 shows a repeated-measures t-test which found that participants’ mean score on COVID-19 knowledge after the training (23.07±2.20) was higher than the mean score before the training (19.50±3.66). This difference was significant, t(133) = -12.68, p < 0.001.

**Table 4.** Pretest and post-test knowledge score.

	Mean±S.D	Mean±S.D	t-test	df	p-value	95% C.I	
						lower	upper
**Pretest**	19.5±3.66						
**Post-test**	23.07±2.20						
**Paired differences**		-3.57±3.26	-12.68	133	˂0.001	-4.13	-3.02

As shown in Table 5, a partial correlation was run to determine the relationship between COVID-19 knowledge and preventive practices towards it, while controlling for attitude. There was a weak partial correlation between knowledge (19.34±3.72) and practice (2.96±1.06) while controlling for attitude (3.28 ± 1.11). However, zero-order correlations showed that there was a statistically significant, moderate, positive correlation between knowledge and practice ( **r**(139) = 0.375, **n** = 141, **p** < .001), indicating that attitude had influence in controlling for the relationship between knowledge and practice.

**Table 5.** Correlation between COVID-19 knowledge and preventive practices towards it.

Correlations				
Control variables		Practice	Knowledge	Attitude
**none** ^a^	Practice correlation significance (2-tailed) df	1.000 0	.375 .000 139	.489 .000 139
	Knowledge correlation significance (2-tailed) df	.375 0.000 139	1.000 0	.441 .000 139
	Attitude correlation significance (2-tailed) df	.489 0.000 139	.441 .000 139	1.000 0
**attitude**	Practice correlation significance (2-tailed) df	1.000 0	.203 .016 138	
	Knowledge correlation significance (2-tailed df	.203 .016 138	1.000 0	

^a^ = Zero-order correlations.

**Figure 1.**
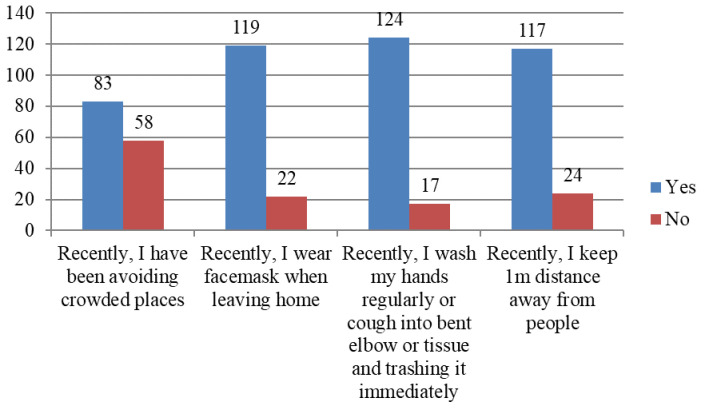
Baseline preventive practices.

Figure 1 shows the assessment of preventive practices toward COVID-19 which was done using (1) avoidance of crowded places, (2) wearing of face masks, (3) regular hand washing with soap and water and coughing into bent elbow or tissue and immediately disposing of it and (4) keeping a social distance of at least 1 meter from people.

A majority (83, 58.9%) reported avoiding crowded places and 119 (84.4%) reported wearing a face mask. When asked about respiratory hygiene, 124 (87.9%) regularly washed their hands with soap and water or coughed into their bent elbows or a tissue. Finally, 117 (83.0%) maintained distance of at least 1 meter when in public places.

**Figure 2.**
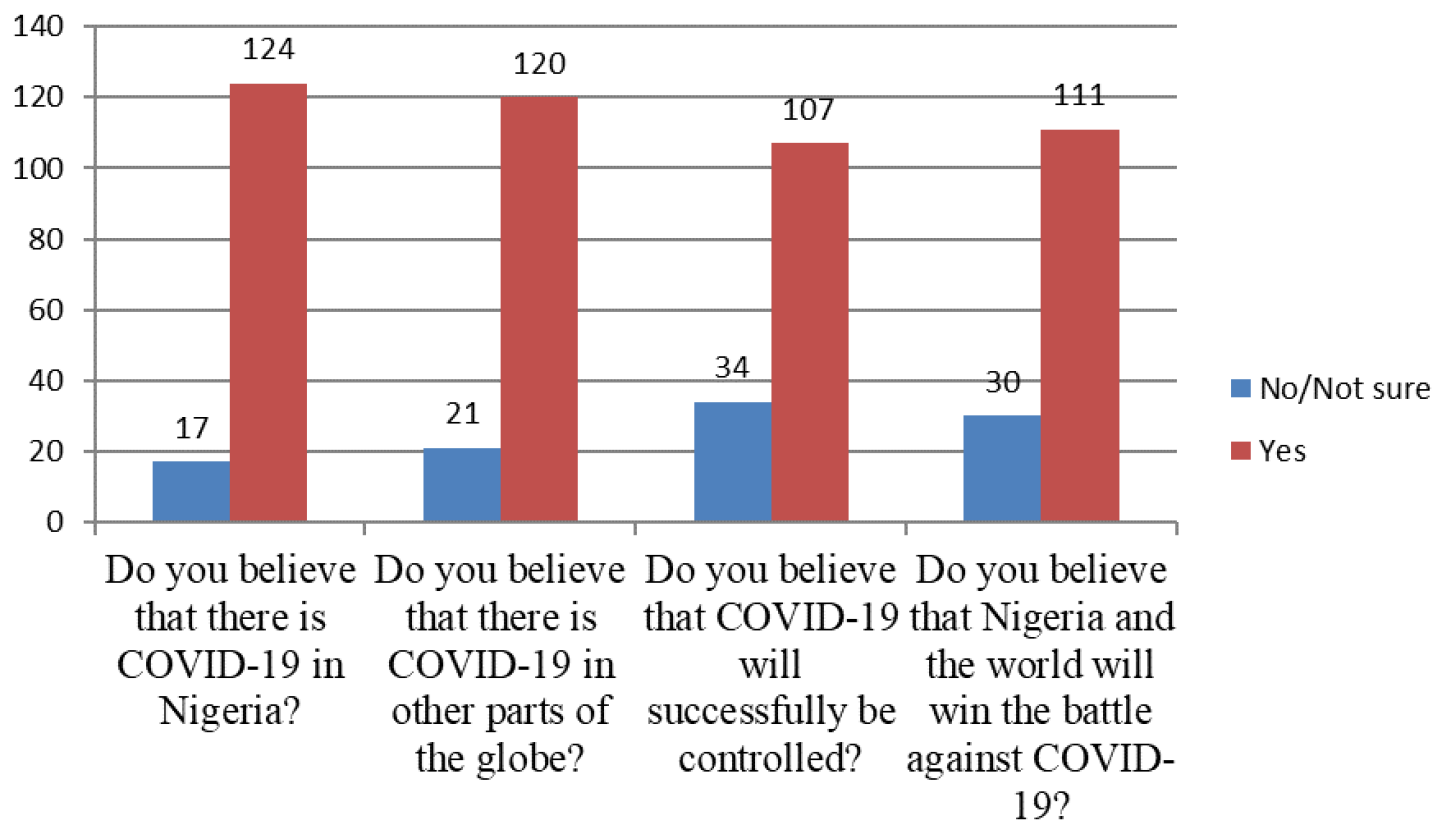
Baseline assessment of attitude of participants towards COVID-19 (N=141).

Figure 2 shows the assessment of attitude toward COVID-19, which found that 124 (87.9) believed there are cases of COVID-19 in Nigeria; 120 (85.1) believed there are cases in other parts of the world; 107 (75.9) believed there will be successful control of the virus, and 111 (78.7) believed that the entire globe will win the battle against the disease.

## Discussion

COVID-19 disease has affected the general population across the globe. The vulnerability risk is higher among the elderly, those with underlying medical pathologies such as diabetes mellitus, obesity, asthma, and systemic hypertension. Low immunity and immune-compromising conditions also increase the morbidity and mortality ( Guan *et al.*, 2020; Zhang *et al.*, 2020).

Given that the prison population is associated with poor access to drugs and health services, and other immune compromising factors, efforts are being made by the entire world, including Nigeria, to prevent and/or reduce the spread of COVID-19 among prison inmates. These efforts include the COVID-19 awareness training organized by the authority of Nigerian Correctional Service. Our study presents the results of the preventive practices and impact of awareness training on COVID-19 knowledge among correctional officers. This is the first COVID-19 awareness intervention study carried out in Nigeria among correctional officers.

### Knowledge about the symptoms, spread, prevention and epidemiology of COVID-19

Our study revealed a high overall knowledge about the disease among the participants, which reflects the findings of previous studies in Egypt and Jordan ( Abdelhafiz *et al.*, 2020; Alzoubi *et al.*, 2020). It has been documented that people who received information about a disease from organized health institutions have better knowledge of the disease than those who obtained information from friends and other informal settings ( Tandi *et al.*, 2018). Most of our participants received COVID-19 information from organized health institutions (NCDC) and multiple sources which include this institution; this may explain the high knowledge level of COVID-19 among them. Nevertheless, the specific question that assessed loss of smell (anosmia) as a symptom of the disease was correctly answered by less than half of the participants. The poor knowledge regarding this specific question may be explained by several factors including the fact that at the time of the study, evidence of loss of smell as a symptom was a recent emergence. This may be further supported by the fact that social media and other informal sources of information erroneously circulated information about the effectiveness of some medicinal products, unapproved by the WHO or any drug regulatory body, against the disease.

A liitle below half of the participants believed that there was vaccine against the disease even when there was none available at the time of the study. Between the time of conducting this study and now, a lot have changed about the disease. One of these changes is that vaccines are now available and the awareness is high ( Uzochukwu *et al.,* 2021).

We found that the awareness training significantly improved the participants’ knowledge about COVID-19 as there was significant evidence that participants had greater knowledge after the awareness training than before.

We also found that there was a significant moderate, positive correlation between knowledge and attitude. Preventive practices also showed significant moderate, positive correlation with knowledge. A similar relationship was documented in Bangladesh and China ( Rahman & Sathi, 2020; Zhong *et al.*, 2020). These associations can be linked to the fact that the wide media coverage of the disease covers aspects of knowledge, attitude, and preventive practices about the disease.

Educational qualification was the only socio-demographic characteristic associated with knowledge and those with higher educational qualifications had more knowledge about the disease, which echoes previous reports in Iran and Nigeria ( Erfani *et al.*, 2020; Okoro *et al.*, 2020a). However, age, source of information, and gender showed no significant relationship with knowledge in our study. Given the wide publicity and awareness of the disease across all ages and gender, it is therefore unsurprising to find no significant relationship between these demographic characteristics and knowledge of COVID-19. 

### Attitudes towards the preventive measures of COVID-19

Our participants generally had a positive attitude towards COVID-19. Similarly, responses to each of the four questions asked to evaluate attitude towards COVID-19 showed that more than four-fifths of the participants believed that there are confirmed cases of the disease in Nigeria, with a similar result being reported about the presence of confirmed cases in other parts of the globe. Three-quarters of the participants believed that the disease will be successfully controlled, and a little above that believed that the world will win the fight against the disease. These results are in accordance with the findings of previous studies in Malaysia and China, in which a positive attitude was reported ( Azlan *et al.*, 2020; Zhong *et al.*, 2020), but differ from the findings in Bangladesh where most of the participants had a negative attitude ( Rahman & Sathi, 2020).

The association between attitude and socio-demographic factors in our study revealed that the belief that there are confirmed cases in Nigeria is significantly associated with the source of information and educational qualification. Furthermore, agreement that there are confirmed cases of COVID-19 in other parts of the world, and that the world will win the fight against the disease showed significant association with educational qualification. This agrees with the findings in China ( Zhong *et al.*, 2020), in which a higher educational qualification was associated with a positive attitude. However, unlike the earlier studies, our study showed no significant association between attitude and age or gender.

### Preventive practices towards the disease

Our study also found an overall high level of preventive practice towards the disease. This reflects the right measures to prevent the spread of the disease and includes wearing face masks, hand washing, avoiding crowded places, and keeping a distance of at least 1 meter away from people.

Avoidance of crowded places was practiced by 58.9% of the participants. The corresponding figures for those that wore face masks, regularly washed their hands, and maintained a 1 meter distance from people were 84.4%, 87.9%, and 83%, respectively. These findings are in consonance with the results in Uganda ( Olum *et al.*, 2020).

Additionally, the practice of hand washing in our study was similar to findings of a Malaysian study ( Azlan *et al.*, 2020), while that of wearing a face mask in public places agrees with a Chinese study ( Zhong *et al.*, 2020).

Considering the findings of our study, there is a need to improve correctional officers’ knowledge via awareness programs, which will further impact positively on their attitude and practices towards the disease. Therefore, these underscore the importance of the Nigerian Correctional Services organizing a nation-wide awareness program for all correctional officers. Furthermore, the poor preventive practice with respect to avoiding crowded places highlights the need for government to enforce stringent measures that will regulate gathering in public places such as markets and even workplaces.

### Limitation

This study was conducted when COVID-19 was relatively a new disease and when the scientific community and the entire world knew little or nothing about it. Presently, there is advanced knowledge of the disease including the availability of various types of COVID-19 vaccines. Similarly, the disease is now well represented in all countries of the world. Hence, some of the concerns this study sought to address (for example, the questions about the availability of COVID-19 vaccine and the one about the presence of confirmed cases of COVID-19 in Nigeria) are now invalid.

## Conclusion

Our study revealed a high level of knowledge, practices and attitude among correctional officers towards COVID-19. Such observations reflect the efforts made by the Nigerian Correctional Service, and the government to sensitize the general population about COVID-19. The findings of this study can be a guide for awareness programs among correctional officers for effective containment of the disease.

## Data availability

### Underlying data

Figshare: CSV data on Attitude and preventive practices towards COVID-19 disease and the impact of awareness training on knowledge of the disease among correctional officers.csv. https://doi.org/10.6084/m9.figshare.12728192.v1 ( Okoro *et al.*, 2020b)

Figshare: Data dictionary. https://figshare.com/articles/Data_dictionary/12728372 ( Okoro *et al.*, 2020c)

### Extended data

Figshare: Questionnaire. https://doi.org/10.6084/m9.figshare.12728375.v1 ( Okoro *et al.*, 2020d)

Data are available under the terms of the Creative Commons Zero "No rights reserved" data waiver (CC0 1.0 Public domain dedication).
