# Supplementary material for: Attitude and preventive practices towards COVID-19 disease and the impact of awareness training on knowledge of the disease among correctional officers
Source: Emerald Open Res. 2022 Jan 21;2:51. doi: 10.35241/emeraldopenres.13839.2 (PMC8830800; doi:10.35241/emeraldopenres.13839.2)
Supplement: Peer review history

# Peer review: Reviewer response for version 2

**DOI:** 10.21956/emeraldopenres.15616.r27977

**Published:** 2022-02-03

**Authors:** P. Ravi Shankar

**Affiliations:** 1 IMU Centre for Education, International Medical University, Kuala Lumpur, Malaysia

The authors have responded to the comments and I am happy to approve this version.

Is the argument information presented in such a way that it can be understood by a non-academic audience?

Not applicable

Could any solutions being offered be effectively implemented in practice?

Not applicable

Is the work clearly and accurately presented and does it cite the current literature?

Partly

If applicable, is the statistical analysis and its interpretation appropriate?

I cannot comment. A qualified statistician is required.

Is real-world evidence provided to support any conclusions made?

Yes

Are all the source data underlying the results available to ensure full reproducibility?

Yes

Is the study design appropriate and is the work technically sound?

Yes

Are the conclusions drawn adequately supported by the results?

Yes

Does the piece present solutions to actual real world challenges?

Not applicable

Are sufficient details of methods and analysis provided to allow replication by others?

Yes

Reviewer Expertise:

COVID-19, Knowledge, attitude, practice, education

I confirm that I have read this submission and believe that I have an appropriate level of expertise to confirm that it is of an acceptable scientific standard.

# Peer review: Reviewer response for version 1

**DOI:** 10.21956/emeraldopenres.14918.r27874

**Published:** 2021-11-23

**Authors:** P. Ravi Shankar

**Affiliations:** 1 IMU Centre for Education, International Medical University, Kuala Lumpur, Malaysia

This is an important study on knowledge, attitude and practice regarding COVID-19 among correctional officers at a specific command in Nigeria before and after an educational intervention.

 The manuscript is mostly well-written and presented. My suggestions for further improvement follow:

- The authors can use a structured abstract and present their findings in a more logical sequence.
- Methods section: Was the questionnaire pre-tested or assessed for ease of understanding before administration? I assume it was administered in English.
- The authors should provide more details about the training program. Who were the facilitators? What were the teaching-learning methods used? What topics were addressed?
- One of the problems with reviewing the manuscript in November 2021 is that many things have changed about the pandemic. The questions like there are cases in Nigeria and there are cases in the world do not seem very relevant now. Also, now we have vaccines and even drugs for treating the condition. The authors should address this limitation in any new version of the article and it would be useful to indicate that future versions of this study will engage with knowledge surrounding vaccines and other treatments developed since the original research was undertaken.
- The authors may have to provide an update about this article and the status of COVID-19 in Nigeria and especially among prisons in the country. A few new references can be added.
- They can provide the questionnaire used in the study as a supplementary file.
- The quality of written English is good.

Is the argument information presented in such a way that it can be understood by a non-academic audience?

Not applicable

Could any solutions being offered be effectively implemented in practice?

Not applicable

Is the work clearly and accurately presented and does it cite the current literature?

Partly

If applicable, is the statistical analysis and its interpretation appropriate?

I cannot comment. A qualified statistician is required.

Is real-world evidence provided to support any conclusions made?

Yes

Are all the source data underlying the results available to ensure full reproducibility?

Yes

Is the study design appropriate and is the work technically sound?

Yes

Are the conclusions drawn adequately supported by the results?

Yes

Does the piece present solutions to actual real world challenges?

Not applicable

Are sufficient details of methods and analysis provided to allow replication by others?

Yes

Reviewer Expertise:

COVID-19, Knowledge, attitude, practice, education

I confirm that I have read this submission and believe that I have an appropriate level of expertise to confirm that it is of an acceptable scientific standard, however I have significant reservations, as outlined above.

# Response

**Published:** 2022-01-10

**Authors:** Johnson Okoro

**Affiliations:** Nigerian Correctional Service, Nigeria

The abstract has been rewritten in a structured way

 Questionnaire was administered in English.

 The program was facilitated by the consultant psychiatrist and head of the medical department of the Nigerian Correctional Service, Enugu State Command, other mental health professionals (psychiatrists and psychologists) and research experts from the CAPIO (Carmelite Prisoners Interest Organization).

 The topics addressed during the training comprised: symptoms of COVID-19, epidemiology and risk factors of COVID-19, disease transmission, and guidelines and preventive measures against it.

 It was an interactive teaching method.

 When the study was conducted, all the questions contained in the questionnaire were relevant at that time. It is true that some of the items in the questionnaire appear to have lost relevance with the passage of time as you correctly noted. However, nothing can be done at the stage of analysis to correct this temporal loss of relevance. Except another study will have to be conducted, which is a part of recommendation for future research in this area.

 All data in respect of this study are with Emerald (the publishing journal).

 Thank you

# Peer review: Reviewer response for version 1

**DOI:** 10.21956/emeraldopenres.14918.r27174

**Published:** 2020-11-02

**Authors:** Felix Bongomin

**Affiliations:** 1 Gulu University, Gulu, Uganda

The present work evaluated the KAP on COVID-19 among correctional officers in Enugu state, Nigeria.

 The manuscript would benefit from the following revisions:

1. "COVID-19 Disease" in the title and elsewhere in the manuscript should be revised to COVID-19, and the disease deleted.
2. The abstract lacks the number of study participants and basic demographic characteristics.
3. The Pre and post tests K assessment should be included in the abstract since it's one of the major objectives of the study.
4. The abstract has no conclusions.
5. Abstract should be organised in such a way that it flows (background, objectives, methods, results and conclusions, even though it is not structured).
6. Introduction. discovered in Wuhan is inappropriate -- first reported.
7. As a result of animal - to human transmission is not corrected... it's a speculation.
8. Table 4 can be substituted with "individual and box and whisker plot " to show a visual display of the K change pre and post tests.

Is the work clearly and accurately presented and does it cite the current literature?

Yes

If applicable, is the statistical analysis and its interpretation appropriate?

Yes

Are all the source data underlying the results available to ensure full reproducibility?

Yes

Is the study design appropriate and is the work technically sound?

Yes

Are the conclusions drawn adequately supported by the results?

Yes

Are sufficient details of methods and analysis provided to allow replication by others?

Yes

Reviewer Expertise:

Clinical Research, Epidemiology , Infectious Diseases

I confirm that I have read this submission and believe that I have an appropriate level of expertise to confirm that it is of an acceptable scientific standard.
